# Micromachined Silicon Platform for Precise Assembly of 2D Multilayer Laue Lenses for High-Resolution X-ray Microscopy

**DOI:** 10.3390/mi11100939

**Published:** 2020-10-15

**Authors:** Wei Xu, Weihe Xu, Nathalie Bouet, Juan Zhou, Hanfei Yan, Xiaojing Huang, Ming Lu, Maxim Zalalutdinov, Yong S. Chu, Evgeny Nazaretski

**Affiliations:** 1 National Synchrotron Light Source II, Brookhaven National Laboratory, Upton, NY 11973, USA; weixu@bnl.gov (W.X.); weihexu@bnl.gov (W.X.); bouet@bnl.gov (N.B.); zhouj@bnl.gov (J.Z.); hyan@bnl.gov (H.Y.); xjhuang@bnl.gov (X.H.); ychu@bnl.gov (Y.S.C.); 2 Center for Functional Nanomaterials, Brookhaven National Laboratory, Upton, NY 11973, USA; mlu@bnl.gov; 3 Naval Research Laboratory, Washington, DC 20375, USA; maxim.zalalutdinov@nrl.navy.mil

**Keywords:** MEMS, microfabrication, multilayer Laue lenses, X-ray microscopy

## Abstract

We report on a developed micromachined silicon platform for the precise assembly of 2D multilayer Laue lenses (MLLs) for high-resolution X-ray microscopy. The platform is 10 × 10 mm^2^ and is fabricated on ~500 µm thick silicon wafers through multiple steps of photolithography and deep reactive-ion etching. The platform accommodates two linear MLLs in a pre-defined configuration with precise angular and lateral position control. In this work, we discuss the design and microfabrication of the platform, and characterization regarding MLLs assembly, position control, repeatability, and stability. The results demonstrate that a micromachined platform can be used for the assembly of a variety of MLLs with different dimensions and optical parameters. The angular misalignment of 2D MLLs is well controlled in the range of the designed accuracy, down to a few millidegrees. The separation distance between MLLs is adjustable from hundreds to more than one thousand micrometers. The use of the developed platform greatly simplifies the alignment procedure of the MLL optics and reduces the complexity of the X-ray microscope. It is a significant step forward for the development of monolithic 2D MLL nanofocusing optics for high-resolution X-ray microscopy.

## 1. Introduction

**Figure 1:**
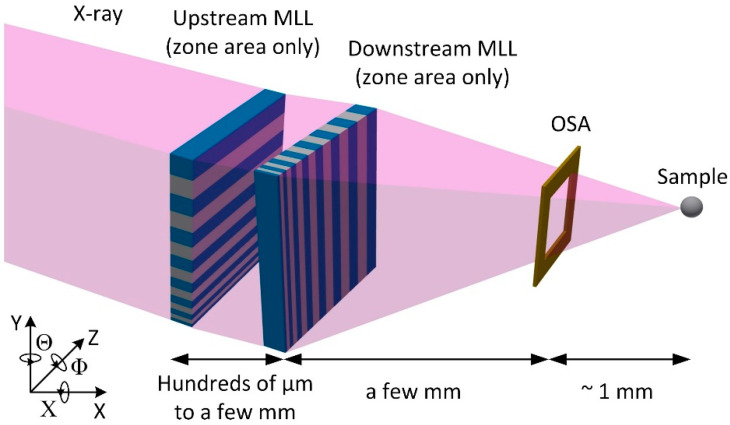
Schematic of multilayer Laue lenses (MLL)-based X-ray microscope. A total of eight degrees of rotational and translational motions is required to perform full alignment of two linear MLLs in a very small envelope.

X-ray microscopy is a powerful characterization tool applied in many scientific fields, such as materials science, biology, environmental science, and energy research [1,2,3,4,5,6,7,8]. Among different types of X-ray focusing optics [9,10,11,12,13,14,15], multilayer Laue lenses (MLLs) have been proposed and used for high-efficiency nanofocusing in the hard X-ray regime [9,16,17]. MLLs are the diffractive optics comprised of layers of alternating materials with different refractive indices (e.g., WSi_2_/Si, W/SiC, and WC/SiC), and they are fabricated via various methods such as magnetron sputtering deposition or pulsed laser deposition [9,18,19,20]. Since MLLs are 1D focusing elements, a pair of MLLs need to be orthogonally aligned with each other to achieve point focusing [21] (Figure 1). There are a total of eight degrees of rotational and translational motions that are required to perform full alignment of two linear MLLs. For example, for a 10 nm diffraction-limited resolution lens, tolerance for orthogonal misalignment should not exceed 0.01°, and the separation between the focal planes of two MLLs should be within the depth of focus (e.g., ~2 µm), in order to preserve the diffraction-limited point focus [22,23]. Once established, alignment must be maintained for the entire duration of an experiment, which spans over many hours or days (especially for tomographic, 3D imaging). Such experimental boundaries define bonding requirements in terms of angular and lateral positions of two lenses with respect to each other. Furthermore, a typical working distance between the order sorting aperture (OSA) and the focal plane does not exceed 1 mm for imaging with 10 nm spatial resolution at 12 keV photon energy. The distance between MLLs and an OSA is less than a few millimeters (Figure 1), making angular adjustments possible only in a tiny spatial envelope. All of these requirements pose significant technical challenges for a microscopy system itself and require an extremely complex and stable instrument. Several sophisticated MLL-based X-ray microscopes have been designed, constructed, and commissioned in recent years [24,25,26,27,28]. For example, a high-resolution scanning hard X-ray microscope has been developed at the Hard X-ray Nanoprobe (HXN) beamline of the National Synchrotron Light Source II (NSLS-II). The HXN endstation utilizes state-of-the-art piezo-mechanical components and provides positioning accuracy down to a few nm when using MLL optics [13,28]. While the HXN instrument works well for many experiments with a point focus of ~10 nm, the alignment of two 1D MLL optics in the current system is a complex procedure that involves eight degrees of motion and requires nm-scale resolution. Moreover, alignment of 1D optics with sub-10 nm resolution may not be feasible due to geometrical constraints of the optics itself. 

Development of a monolithic 2D MLL nanofocusing optics could minimize the degrees of the nanoscale motion needed for MLL alignment, reduce the complexity of an instrument, and enable sub-10 nm focusing experiments in the hard X-ray regime. There has been an increasing number of efforts in the past few years, targeting the development of the monolithic 2D MLL optics [23,29,30,31,32]. For example, S. Niese and A. Kubec et al. glue-bonded two MLLs together and demonstrated sub-100 nm resolution during full-field imaging and ~43 nm 2D focusing during ptychography experiments [29,32]. W. Xu and E. Nazaretski et al. explored UV adhesive-assisted direct bonding and demonstrated 12 × 24 nm^2^ point focus [31]. However, the uncontrollable stress of the adhesives used for bonding makes the direct bonding approach extremely challenging for precise angular and lateral position control. It is especially true in the case of wedged MLLs when parameters of the lenses are tailored to a specific photon energy, and astigmatism cannot be compensated by changing the X-ray energy without compromising efficiency. The adhesives may also cause potential contamination of the MLL optics. 

Recently, we reported a new 2D MLL nanofocusing optics developed on a micromachined silicon platform [33]. We demonstrated a point focus of ~10 nm in size using the developed 2D MLL optics. Here, we focus on the micromachined silicon platform used for the precise assembly of 2D MLL optics. We introduce the design and working mechanism of the platform, as well as the microfabrication process. We characterize the platform regarding MLLs assembly, position control, repeatability, and stability. The results show the micromachined platform provides a simple and accurate approach for the precise assembly of MLL optics with varying sizes and optical parameters. It significantly contributes to the development of MLL-based high-resolution X-ray microscopy and enables a path toward sub-10 nm resolution imaging in the hard X-ray regime.

## 2. Results

### 2.1. Design of the Micromachined Silicon Platform

**Figure 2:**
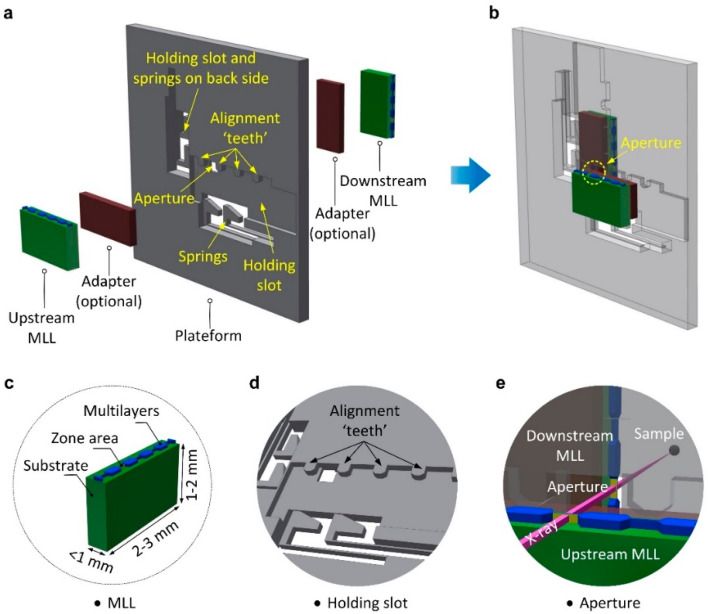
Schematic of 2D multilayer Laue lenses (MLLs) assembled using a micromachined silicon platform. (**a**) A silicon platform shows the key features, including holding slots, alignment “teeth”, springs, and an aperture. (**b**) 2D MLL optics assembled using the platform. (**c**) Schematic of a linear MLL. (**d**) The details of the holding slot on the platform. (**e**) A magnified view shows that an X-ray beam passes through the aperture and illuminates two overlapping MLLs.

Figure 2a,b illustrate a schematic of the 2D multilayer Laue lenses (MLLs) assembled using a micromachined silicon platform. The 1D MLL optics used in our work was fabricated via magnetron sputtering deposition on a silicon substrate and further sectioned by reactive-ion etching followed by focused ion beam (FIB) milling [33]. As shown in Figure 2c, the MLL includes a silicon substrate, deposited Si and WSi_2_ multilayers, and an FIB-polished zone area. The overall dimensions of the MLL optics are 2–3 mm long, 1–2 mm high, and less than 1 mm thick in general. The Si platform is designed based on the dimensions and optical parameters of available MLLs. It has dimensions of ~10 × 10 × 0.5 mm^3^, fabricated on a ~500 µm thick silicon wafer. On each side of the platform, there is a holding slot, which includes four alignment “teeth” and two cantilever-type springs (the details are shown in Figure 2d). Two holding slots on the front and back sides of the wafer are aligned orthogonally with respect to each other. At the center of the platform, there is an aperture, where silicon is etched through. To mount MLLs onto the platform, each MLL is placed into the loading zone at the right corner of a holding slot and then is slid to the location near the aperture. Slopes are designed at the corner of each tooth and the spring head for slip-fitting MLLs in the holding slots. Considering the different dimensions of the MLL optics, adapters are used if the sizes of MLLs do not fit the holding slot. In this particular case, the MLL is attached to an adapter, and then the combined assembly of the MLL and adapter is mounted onto the template. Once the MLLs are mounted, the springs push on the bottom surface of the MLLs (or adapters) so that the MLL structure is positioned against the alignment teeth and secured through the force exerted by the Si springs. The active zones area of two MLL optics overlaps at the aperture, where the X-rays pass through to illuminate both MLLs (Figure 2e). The details of the design of the platform regarding the angular and lateral position control, springs, and aperture are discussed in the following.

#### 2.1.1. Angular Position Control

**Figure 3:**
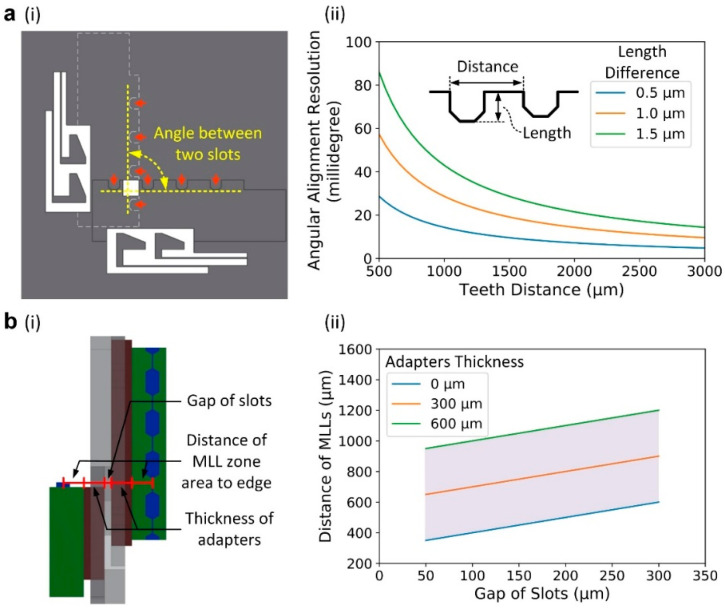
Angular and lateral position control of MLLs. (**ai**) Angle between two holding slots, defined by the position of alignment teeth (indicated by red arrows). (**aii**) The dependence of the angular alignment resolution on the morphology of the alignment teeth. (**bi**) The separation distance between two MLLs is the sum of the gap between two slots, the distance of MLL zone area to the edge, and the thickness of adapters. (**bii**) The change of the distance between MLLs with the gap of slots and the thickness of adapters (the distance of MLL zone area to the edge is set to 150 µm as an example).

The angular position of the MLLs mounted onto the platform is controlled and adjusted through a series of prefabricated alignment teeth, as shown in Figure 3a(i). Several alignment teeth are placed along the edge of a holding slot to precisely define the location of an MLL within a platform. By changing the length and distance of adjacent alignment teeth, one can adjust the angle between two slots inside the platform and, consequently, the orthogonality of MLLs mounted in the slots. For example, if the length difference of adjacent alignment teeth is 1 µm and the distance between them is 1000 µm, the angle between two slots deviates by 57 millidegrees from a perfect 90° configuration. In this work, we have fabricated a set of templates with the angle between two slots gradually changing from 89.7° to 90.3° with a step of ~50 millidegrees. Half of the angle change step defines the resolution of templates for the angular alignment (i.e., ~25 millidegrees in this work). Figure 3a(ii) illustrates the dependence of the angular alignment resolution on the morphology of the alignment teeth. The resolution becomes smaller with the increase in the spacing between adjacent teeth and the decrease in the length difference between teeth. By choosing a platform with the most appropriate angle between two slots, one can reduce angular misalignment between two MLLs induced by various factors such as surface imperfections of MLLs, artifacts of the microfabrication process, and the use of adapters.

#### 2.1.2. Lateral Position Control

The separation distance of mounted MLLs along the X-ray beam direction must mimic the focal length differences of individual MLLs in order to achieve an astigmatism-free point focus. As mentioned before, the separation distance between two lenses must be controlled within 2 µm range (depth of focus) for 10 nm focus size at 12 keV photon energy [23]. By using the micromachined platform, one can adjust the distance between two MLLs by changing the depth of etched holding slots (i.e., changing the gap between two slots) or the thickness of adapters (Figure 3b(i)). For example, if the distance of the MLL zone area to the edge is ~150 µm, by changing the gap between two slots from 50 to 300 µm and meanwhile changing the total thickness of two adapters from 0 (i.e., no adapter used) to 600 µm, one can change the distance between two MLLs from 350 to 1200 µm (Figure 3b(ii)). The resolution of the etching depth is affected by the etching rate, which could change from a few micrometers to sub-micrometers per minute through a Bosch or cryogenic etching process [33]. By changing the etching rate or time, one can keep the error of separation distances down to sub-micrometers, therefore controlling the position of the MLLs along the X-ray beam with high accuracy.

#### 2.1.3. Silicon Springs and Aperture

**Figure 4:**
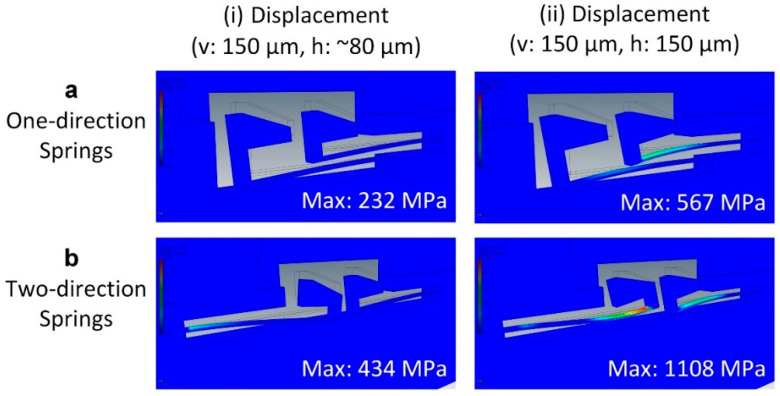
Deformation and stress applied to the template springs with the loading of MLLs. The maximal Von Mises stress applied to (**a**) one-direction springs and (**b**) two-direction springs in two cases: (**i**) a vertical displacement of 150 µm with a simultaneous horizontal displacement of ~80 µm; (**ii**) a vertical displacement of 150 µm with a horizontal displacement of 150 µm.

Cantilever-type silicon springs have been used to secure the position of linear MLLs mounted onto the platform. We have designed two types of cantilever springs: one-direction springs and two-direction springs (Figure 4). Each spring is 2850 µm long and 20–40 µm wide. We have compared the stress applied to two types of springs with the mounting of MLLs to ensure the mechanical robustness of the platforms. As shown in Figure 4, when the displacement of spring heads in the vertical direction (i.e., the direction perpendicular to the holding slot) increases to 150 µm with the mounting of MLLs, the simultaneous horizontal displacements are ~80 µm, and the maximum Von Mises stresses applied to one-direction and two-direction springs are 232 and 434 MPa, respectively (spring width: 40 µm, thickness: 200 µm). The stress applied to the springs is substantially lower than the yield strength of silicon (i.e., 7 GPa). Further, the stress applied to the two-direction springs is higher than that of the one-direction springs. When the MLL slides onto the spring head during the mounting process, it may result in an extra displacement of springs in the horizontal direction due to the surface friction. In this situation, if the horizontal displacement of the spring heads increases to 150 µm while the vertical displacement is 150 µm, the maximum stress applied to two kinds of springs is much larger than what has been described in the previous cases. It is more significant for two-directional springs, in which the maximal Von Mises stress reaches 1108 MPa. Based on the simulation results, one-directional springs have been used in this work.

The aperture at the center of the platform yields the X-rays to pass through and illuminate both MLLs. Since partial MLLs that have been used in this work are an off-axis X-ray diffractive optics, the X-ray beam bends after it goes through an upstream MLL by less than a degree, yielding a maximum lateral displacement of ~17 µm (from a direct path), assuming the downstream MLL is located 1000 µm away from the upstream one. Bearing that in mind and considering the additional tilting of MLLs required to satisfy Bragg conditions, we have designed an aperture of 500 × 500 µm^2^ allowing the maximum tilting angle of ±14 degrees without blocking the X-ray beam.

### 2.2. Microfabrication of Silicon Platform

**Figure 5:**
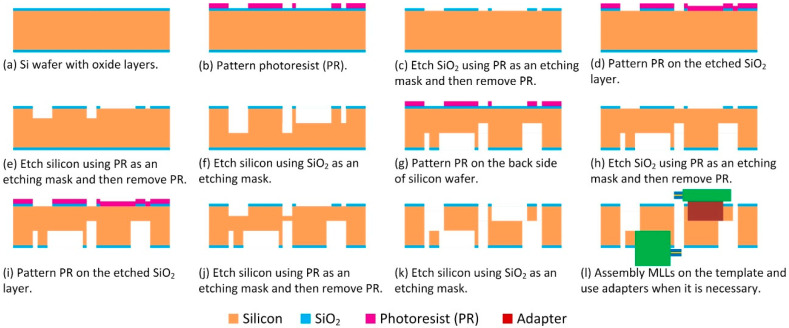
Fabrication process of the silicon platform.

The silicon platform has been fabricated on ~500 µm thick 4-inch silicon wafers. The microfabrication process includes multiple steps of photolithography and deep reactive-ion etching (DRIE), as depicted in Figure 5. In particular, a silicon wafer with a 3 µm thick oxide layer (Figure 5a) was first spin-coated with photoresist (AZ4620, MicroChemicals, Ulm, Germany) at 4000 rpm for 40 s, followed by baking at 110 °C for 90 s. The wafer was then exposed using a mask aligner (MA-6, SUSS MicroTec, Garching, Germany) in soft-contact mode, and developed in developer solution (AZ400K, 1:3, MicroChemicals, Ulm, Germany) (Figure 5b). The patterned photoresist AZ4620 was then used as an etch mask to etch the SiO_2_ layer through a dry etching process (Oxford PlasmaLab 100, Oxford Plasma Technology Inc., Bristol, UK) (Figure 5c). After that, the substrate was spin-coated with AZ4620 again using the second photomask, followed by a similar exposure and development process (Figure 5d). In the following, the patterned photoresist and SiO_2_ served as etching masks to etch the silicon substrate through two steps of the Bosch etching process (Oxford PlasmaPro System 100 Cobra, Oxford Plasma Technology Inc.) (Figure 5e,f). Table 1 shows the recipes of the Bosch process for silicon etching. The etching rate is ~3 µm/min. The same photolithography and etching process then proceeded on the backside of the silicon substrate (Figure 5g–k). For the etching-through process (Figure 5k), the wafer was cut into small chips, which were then attached to a carrier wafer for etching. After the whole process, holding slots and silicon springs were fabricated on both sides of the wafer, and an aperture was etched at the center. The fabricated platform was then ready to be used for the assembly of MLLs (Figure 5l).

**Figure 6:**
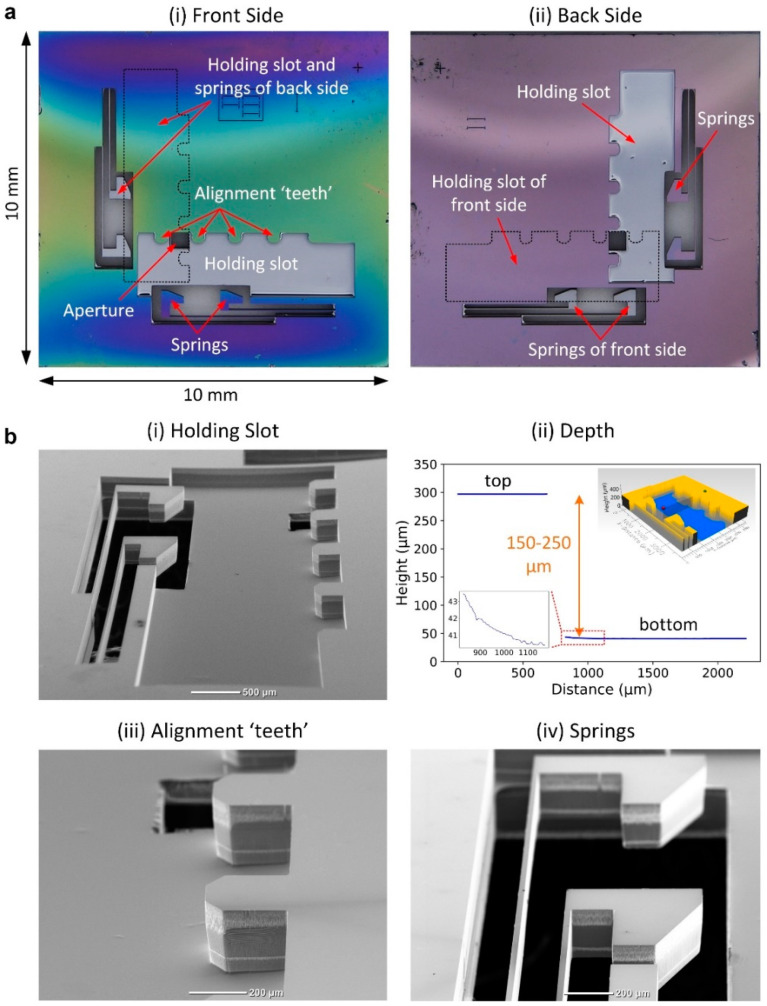
Micromachined silicon platform. (**a**) The optical images of a micromachined silicon platform. (**i**) The front side, (**ii**) the back side. (**b**) The SEM images showing the details of the (**i**–**ii**) holding slot, (**iii**) alignment teeth, and (**iv**) springs of the platform.

Figure 6 shows an example of the microfabricated silicon platform. The overall lateral dimensions of the platform, as we have mentioned before, are ~10 × 10 mm^2^. There is an etched holding slot, along with alignment teeth and springs on each side of the platform. Two holding slots are aligned orthogonally to each other (Figure 6a). The surface of etched holding slots is smooth (Figure 6b(i)), and the roughness (*S_a_*) is less than 0.15 µm (Profilm3D, Filmetrics, San Diego, CA, USA). The depths of slots are etched according to the used MLLs, and most of them are between 150 and 250 µm (Figure 6b(ii)). There is no remarkable change in etching depth in most of the holding slot areas. However, it should be noted that, in the area near the slot edge, the etched surface’s profile is gradually changed for a few micrometers, as shown in the inset. Considering the influence of the surface profile on the lateral position of the assembled MLLs, we used an interferometer to measure the actual separation distance between assembled MLLs. The slot will be etched further until the measured result agrees with the target. Since the silicon is etched for ~0.7 µm in each etching cycle of the Bosch process, one can precisely adjust the depth of slots with a resolution better than 1 µm by changing the number of etching cycles. The alignment teeth have been etched along the side of the holding slot and show nearly vertical sidewalls (Figure 6b(iii)). We have fabricated a set of templates with the length difference of adjacent teeth changing from −3 to 3 µm with a step of 1 µm to adjust the angle between mounted MLLs. The functionality of alignment teeth on the angular position control will be discussed in the characterization section of the manuscript. The silicon springs are etched on the other side of the slot. As shown in Figure 6b(iv), the thickness of springs is ~200 µm, and they are well retained after deep etching. The surface of the slope on the spring head and alignment teeth is relatively smooth, which is beneficial for the sliding of MLLs into position. The distance between the top of spring heads to the alignment teeth is 1.35 mm, and the width of slots is 1.6 mm. Therefore, the platform could directly accommodate MLLs with a height between ~1.4 and 1.6 mm. For MLLs that could not be directly mounted, an adapter could be used. It should be noted that the dimensions of slots, alignment teeth, and springs can be re-designed according to particular applications.

### 2.3. Characterization of Platform

We have characterized the platform regarding MLLs assembly, position control, repeatability of installation, and stability of the assembly. Here, we have used silicon substrates of MLLs (which have similar dimensions to the actual MLLs) as dummy samples for characterization, since actual MLLs are extremely valuable and could not be accidentally damaged due to multiple handling steps.

#### 2.3.1. MLLs Assembly

**Figure 7:**
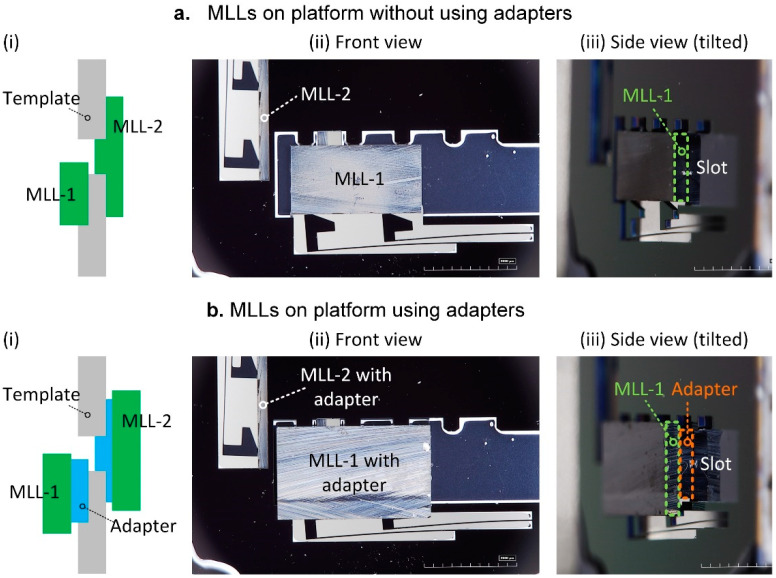
Assembly of dummy MLL samples onto the platform. (**a**) Without using adapters, (**b**) using adapters.

We have tested the assembly of dummy MLL samples onto the platform for two cases: using intermediate adapters and without adapters. Figure 7a shows an example of MLLs mounted directly without using an adapter. With the loading of the MLL sample, the springs bent and remained unbroken with a vertical displacement of 150 µm (the height of the dummy MLL sample is 1.5 mm). The side view (Figure 7(aiii)) indicates that the MLL is mounted well in the slot. Figure 7b illustrates a case where we have used two adapters for the assembly of MLLs that do not fit into the holding slot directly. In this case, we first attach MLLs to the adapters. Since adhesives can be applied to the areas of MLLs which are far away from the active zones area, contamination of the multilayers themselves can be ruled out. The complete MLL/adapter assembly has been installed into the platform slot. For example, Figure 7b demonstrates two MLLs with a height of 2 mm mounted onto the platform by using adapters, while the width of the slot is only 1.6 mm. This approach enables accommodating MLLs with different dimensions without re-designing of a complete platform.

#### 2.3.2. Mutual Orthogonality of Linear MLL Optics

**Figure 8:**
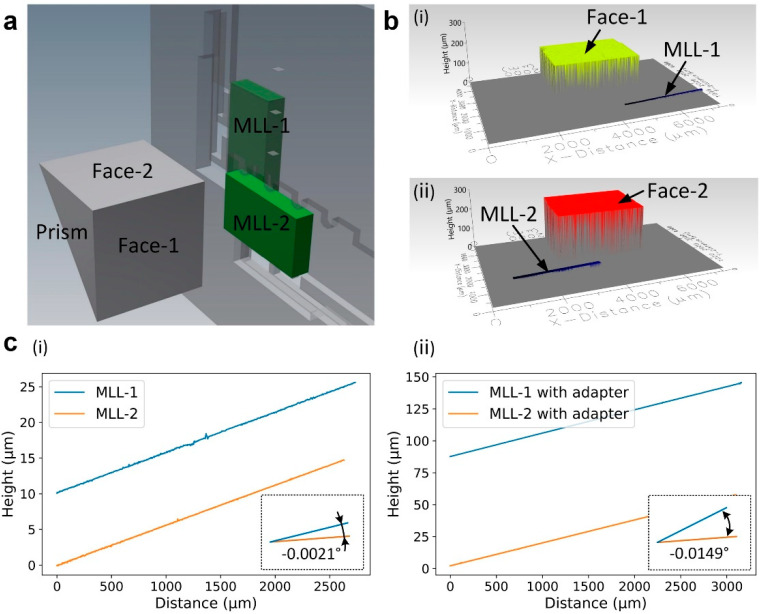
The orthogonality of MLLs assembled onto the micromachined platform. (**a**) A right-angle prism has been used as a reference for the measurement of orthogonality. (**b**) The surface profiles of dummy MLL samples and the reference prism. (**c**) The changes in the heights of MLL surfaces with respect to the prism surfaces. The insets show the angles between two lines, which indicate the deviation of the angle between two MLL surfaces from 90°. (**i**) MLLs directly assembled onto the platform, (**ii**) MLLs assembled onto the platform using adapters.

We have studied the orthogonality of dummy MLL samples assembled onto the platform. For the measurement of orthogonality, we used a right-angle prism (N-BK7 high-tolerance right-angle prism, Edmund Optics, NJ, USA; angle tolerance: 4 millidegrees) as a reference. As shown in Figure 8a, the reference prism has been placed near the MLLs, with each face roughly aligned with the top surface of an MLL sample. Then, we measured the surface profiles of each MLL and the corresponding prism surface using a white-light interferometer (smartWLI compact, GBS mbH, Germany) (Figure 8b). Figure 8c shows the height change of each MLL surface with respect to the prism surface, and the angle between the two lines indicates the deviation of the angle between two MLL surfaces from perfect 90°. For example, for a pair of MLLs that are directly mounted on a platform with a slot angle of 90° (i.e., the angle between two slots is 90°), the deviation of the angle between two MLL surfaces from 90° is −2.1 millidegrees (i.e., the angle between two MLL surfaces is 89.9979°). Figure 8c(ii) shows another example when two MLLs were assembled onto the platform using two adapters. In this case, we selected a platform where the angle between the two slots is 90.1°. This design corrects the small angular misalignment induced by the adapters. The angle deviation of two assembled MLLs from 90° is −14.9 millidegrees (i.e., the angle between two MLL surfaces is 89.9851°). The results demonstrate that the mutual angular misalignment of assembled MLLs, regardless of adapter/non-adapter configuration, can be controlled well within the designed accuracy (i.e., ~25 millidegrees in this work). It should be noted that the angular position can be controlled with higher accuracy, if necessary, through the re-design of a platform according to the requirements of a particular application.

The uncontrolled errors in angular alignment may be attributed to several factors. First, the accuracy of the backside alignment is 1 µm for the mask aligner we used (SUSS MicroTec MA6 mask aligner), which could give an angular error of a few millidegrees. The deep etching process may also result in some errors, since the defects or residuals formed on the sidewall of alignment teeth during etching may affect the position of assembled MLLs aligned against the alignment teeth. Besides, the angular error of the reference prism used in this work is 4 millidegrees, which defines the accuracy limit of the measurements.

#### 2.3.3. Separation Distance along the X-ray Beam Direction

**Figure 9:**
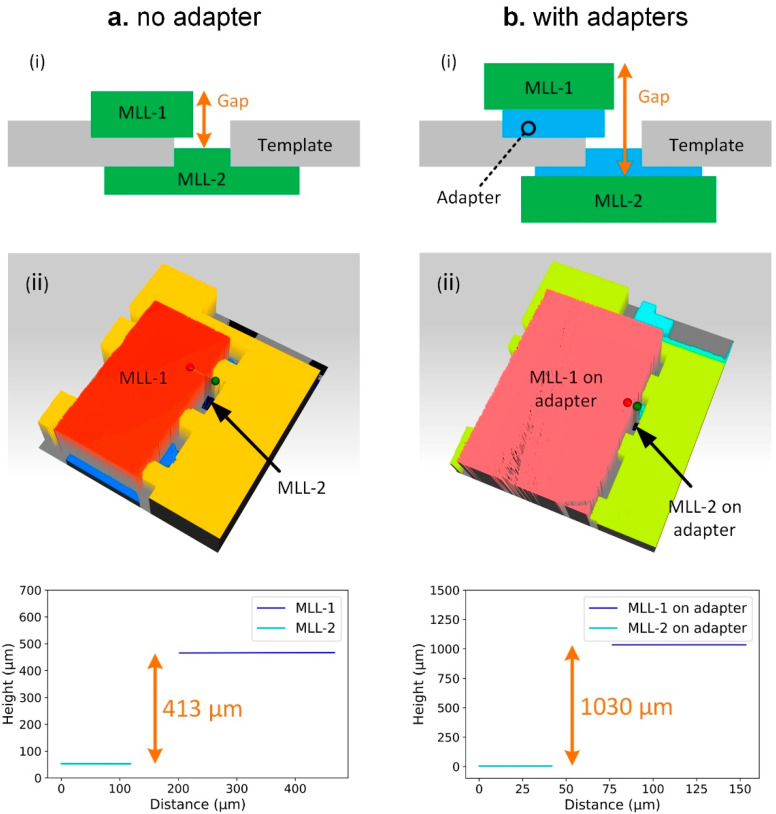
The separation distance between individual MLLs assembled onto the micromachined platform. (**a**) MLLs directly assembled onto the platform. (**b**) MLLs assembled onto the platform using two adapters.

There are two ways one can adjust the separation distance between assembled MLLs when using the platform. First, one can change the etched depth of the holding slots. In this work, we etched the holding slots according to the focal length of individual MLLs and their specific linear dimensions, to have their focal planes overlap after completion of assembly. Since the holding slots were etched through a Bosch process with ~0.7 µm increments per etching cycle, one can adjust the distance between MLLs with a sub-micrometer resolution by changing the etching cycles or time. Second, one can change the distance between MLLs by using various adapters. As shown in Figure 9a, a pair of dummy MLLs of 300 µm thick were directly mounted onto a template, and their distance was 413 µm (Profilm3D, Filmetrics, CA, USA). In another example, by using two adapters of ~300 µm thick, one on each side, the distance between MLLs increased to 1030 µm (Figure 9b). The thickness of adapters can also be modified through the microfabrication process yielding more flexibility to accommodate MLLs with different focal lengths.

#### 2.3.4. Repeatability of Installation and Stability of the Assembly

**Figure 10:**
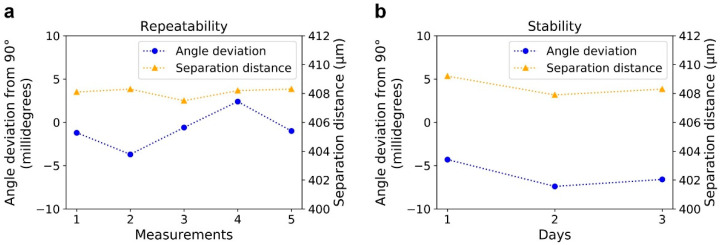
The repeatability and stability of MLLs assembled onto the micromachined platform. (**a**) The changes in the angle and separation distance of assembled MLLs in five consecutive loading/unloading processes. (**b**) The changes in the angle and separation distance between assembled MLLs in 3 days.

We have also studied the repeatability of the installation and stability of MLLs when completely assembled on the micromachined platform. As shown in Figure 10a, during five consecutive loading/unloading processes onto the same platform followed by a characterization measurement, the angle between two MLLs changed by less than 6 millidegrees, and the separation distance along the beam direction changed by less than 1 µm. Further, the changes of angular and lateral positions of assembled MLLs in a three-day time period were less than 3 millidegrees and 1 µm, respectively. The observed instability may occur in that the position of assembled MLLs had a slight change due to the handling in the measurements or environmental vibration. It may also be affected by the measurement error. These results are in the range of our alignment requirements, demonstrating good repeatability and stability of the developed platform.

## 3. Discussion

There are several advantages of using the developed micromachined platform for further developments of 2D MLL optics. First of all, one can precisely control the orthogonality and separation distances between individual MLLs which are defined by the high accuracy of the microfabrication processes. The accuracy can be further improved in case angular and separation requirements become more stringent (a few approaches are discussed as an example in the following paragraph). Second, the micromachined platform is relatively small (~10 × 10 mm^2^), and the angular adjustments needed to satisfy Bragg conditions are very modest, making the required space envelope very compact. It makes it easier to integrate the developed platform into an X-ray microscope and reduce the number of nanoscale motions needed to perform full optics alignment. Furthermore, the assembly of MLLs onto the platform is straightforward and does not involve any complex procedures. The developed 2D MLL platform is somewhat similar to a well-known Fresnel zone plate optics, thereby offering compatibility with many conventional, zone plate-based microscopy systems. We believe our work is an important advancement that will allow broader applications of the MLL optics in the hard X-ray microscopy community.

In recent years, a number of efforts have been made to achieve sub-10 nm point focus in the hard X-ray regime [34]. There are several ways the developed platform can be used and enable nanofocusing below 10 nm. First, as shown in Figure 3a(ii), one can improve the angular alignment resolution by reducing the length difference or enlarging the distance between adjacent teeth. For example, by changing the length difference of teeth to 0.5 µm and the distance between them to 2900 µm, one can improve the resolution to 5 millidegrees, a requirement to achieve 5 nm point focus. Second, one can adjust the bottom side alignment (BSA) process of photolithography when fabricating the platform. By changing the orthogonality of the alignment process, one can further improve the angular alignment resolution. Third, to push the angular accuracy even further, one can use the focused ion beam (FIB) to modify the alignment teeth as well as the clamping point of the adapter. FIB technology allows modifying the microstructures with a nanometer-level resolution, which can achieve the angle control between MLLs with a resolution of ~1 millidegree. In addition, the development of a micromachined platform with an active angle alignment is another promising approach for high-resolution X-ray nanofocusing.

## 4. Conclusions

In summary, we have developed a micromachined silicon platform for the precise assembly of 2D MLL X-ray nanofocusing optics. In this work, we have introduced the design and working mechanism of the developed platform and provided details of the microfabrication process. Developed platforms have been characterized with respect to assembly, MLL position control, mounting repeatability, and long-term stability. The angular misalignment of the assembled MLLs is in the range of the designed accuracy of the platform, down to a few millidegrees. The separation distance between MLLs can be changed from ~400 to more than 1000 µm by using adapters. The position of MLLs assembled onto the platform also shows good mounting repeatability and long-term stability. The developed platform provides a simple approach for the precise assembly of a variety of MLLs with different dimensions and optical parameters. We believe our work marks a significant step forward for the development of monolithic 2D MLL nanofocusing optics for high-resolution hard X-ray microscopy.

## Figures and Tables

**Table 1:** The Bosch process conditions.

Parameters	Etching	Deposition
Cycle time (s)	8	5
C_4_F_8_ gas flow (sccm)	1	100
SF_6_ gas flow (sccm)	100	1
Pressure (mTorr)	30	20
ICP power (W)	1750	1750
RF power (W)	40	5
Temperature (°C)	10	10
